# Functional expression of complement factor I following AAV-mediated gene delivery in the retina of mice and human cells

**DOI:** 10.1038/s41434-021-00239-9

**Published:** 2021-03-10

**Authors:** Anna K. Dreismann, Michelle E. McClements, Alun R. Barnard, Elise Orhan, Jane P. Hughes, Peter J. Lachmann, Robert E. MacLaren

**Affiliations:** 1 Gyroscope Therapeutics Limited, Stevenage Bio-Science Catalyst, Stevenage, UK; 2 Nuffield Laboratory of Ophthalmology, University of Oxford, Oxford, UK; 3 Department of Veterinary Medicine, University of Cambridge, Cambridge, UK; 4 Oxford Eye Hospital NIHR Biomedical Research Centre, Oxford, UK

**Keywords:** Immunotherapy, Immunological disorders

## Abstract

Dry age-related macular degeneration (AMD) is characterised by loss of central vision and currently has no approved medical treatment. Dysregulation of the complement system is thought to play an important role in disease pathology and supplementation of Complement Factor I (CFI), a key regulator of the complement system, has the potential to provide a treatment option for AMD. In this study, we demonstrate the generation of AAV constructs carrying the human CFI sequence and expression of CFI in cell lines and in the retina of C57BL/6 J mice. Four codon optimised constructs were compared to the most common human CFI sequence. All constructs expressed CFI protein; however, most codon optimised sequences resulted in significantly reduced CFI secretion compared to the non-optimised CFI sequence. In vivo expression analysis showed that CFI was predominantly expressed in the RPE and photoreceptors. Secreted protein in vitreous humour was demonstrated to be functionally active. The findings presented here have led to the formulation of an AAV-vectored gene therapy product currently being tested in a first-in-human clinical trial in subjects with geographic atrophy secondary to dry AMD (NCT03846193).

## Introduction

Age-related macular degeneration (AMD) is the most common cause of blindness amongst the elderly in the industrialised world, affecting approximately 36 to 40 million people globally [1]. While the early stages of the disease are characterised by slight disturbances in vision, such as blurred or distorted vision, late stage forms can be associated with severe sight loss that has a dramatic impact on the lives of affected individuals. Late stage AMD is divided into wet AMD and geographic atrophy (GA), the former currently being treated with anti-VEGF therapeutics, while for the latter there is currently no approved medical treatment. The exact molecular pathways leading to disease are not yet identified; however, it is established that AMD is a complex disease and that genetic predisposition together with environmental and demographic factors play a significant role [1]. The complement system, a major effector arm of innate and adaptive immunity, is thought be involved in disease pathology. Complement proteins are accumulated in drusen, one of the main hallmarks of early AMD, and Bruch’s membrane; plasma, aqueous or vitreous humour levels of complement activation fragments C3a, C3dg, Bb and C5a are increased in AMD patients [2–4]. Genome wide association studies (GWAS) further revealed an association of disease with key regulatory proteins of the complement system [5, 6]. Moreover, oxidative stress was linked to AMD through formation of neoepitopes that bind to autoantibodies capable of activating complement [7, 8]. The accumulation of complement proteins may act as a depot of continual complement stimulation by triggering local production of inflammatory mediators and attracting leukocytes that further augment the local inflammatory state, driving AMD pathology. Mutations in Complement C3, Complement Factor I (CFI), Complement Factor H (CFH) and Complement Factor B (CFB) genes strongly correlate with the likelihood of developing AMD, with CFI highlighted as particularly significant [5]. These findings have since been confirmed in further independent studies and low serum CFI levels associated with the presence of rare CFI variants were found to be associated with a much higher risk of developing advanced AMD (P = 5.6E-05) [9, 10]. Together, these findings implicate loss of complement regulatory control in AMD pathogenesis.

**Fig. 1:**
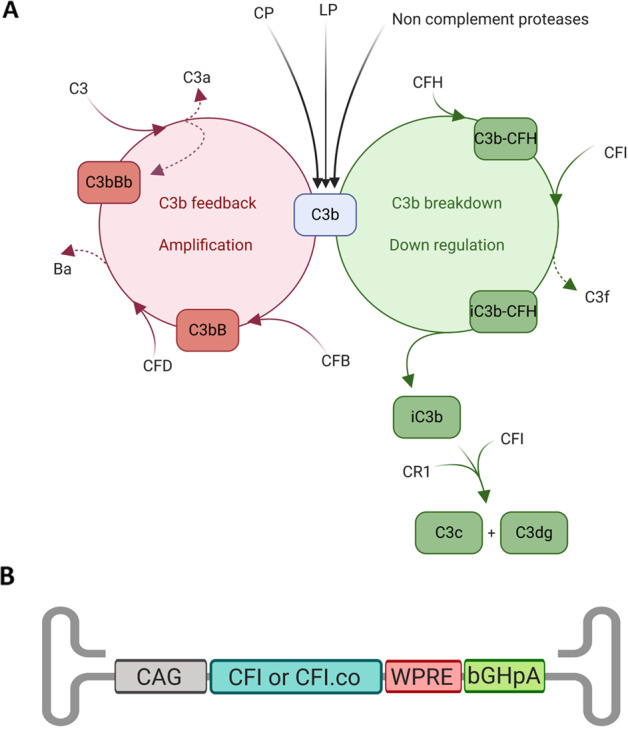
Schematic diagram of the role of CFI in alternative pathway regulation and AAV design. **A** CFI is a key regulator of the alternative pathway and degrades C3b into iC3b and C3dg in the presence of cofactor. Figure adapted with permission from [12]. **B** Schematic diagram of AAV2 backbone coding sequence. AAV = adeno-associated virus, bGHpA = bovine growth hormone poly adenylation sequence, bp = base pair, C3 = Complement C3, CAG = chicken b-actin promoter with a CMV enhancer and a β-globin intron, CFB = complement factor B, CFD = complement factor D, CFH = complement factor H, CFI = complement factor I, CR1 = Complement Receptor 1, WPRE = Woodchuck hepatitis virus post-transcriptional regulator element. Biorender.com was used to generate Fig. 1A, and B.

The complement system is a critical component of the immune system and can be activated via the classical, lectin, or alternative pathway. The first two pathways are activated by binding of their recognition molecules to IgG or IgM immune complexes, C-reactive protein, pentraxin 3, RNA, DNA, extracellular matrix proteins, altered self-structures such as beta-amyloid, apoptotic or necrotic cells, microorganism derived ligands, like LPS or carbohydrate structures on microorganisms or altered self-cells [11]. The alternative pathway (AP) can be activated on protected surfaces [12] but mainly acts as a powerful amplification mechanism of the other two pathways. It depends on the so-called ‘tick-over’ mechanism (more recently described in [13]) and is initiated by binding of CFB to C3b which forms the AP C3 convertase, C3bBb, that cleaves C3 into C3b. The resulting C3b can form another C3 convertase with CFB and thereby creates a positive feedback amplification mechanism that leads to rapid multiplication of C3b molecules and complement effector proteins. The initial C3b is generated by classical or lectin pathway activation or by activated factors of the coagulation, contact activation and fibrinolysis systems (reviewed in [14]). Subsequent deposition of C3b on surfaces happens rapidly and is indiscriminatory, targeting healthy self, altered self or foreign cells alike; however, binding of CFH or properdin (required for stabilisation of the short lived AP C3 convertase) enables the AP to distinguish between target surfaces [15, 16]. On a healthy self-surface, CFH binding predominates and acts as a cofactor to CFI-mediated cleavage of C3b, whereas on foreign or altered self surfaces, properdin binding enables assembly of C3 convertases that amplify initial complement activation. If C3b is not deposited on a surface but in the fluid phase, down-regulation by CFH binding and CFI cleavage is heavily favoured, with the generation of fluid-phase iC3b as an inactive end product [17]. CFI is a key enzyme of the AP: in the presence of a cofactor (CFH, Membrane Cofactor Protein, or Complement Receptor 1), it cleaves C3b into iC3b and further into C3dg. Both cleavage reactions are essential to prevent positive amplification of the alternative pathway (by degradation of C3b into iC3b) and to degrade pro-inflammatory iC3b into inert C3dg (Fig. 1A). Raising the concentration of CFI in serum was found to inhibit initiation of the AP [18]. The AP performs a continuous surveillance function and is under strict control of plasma and membrane bound complement regulator proteins. Due to the rapid self-amplification mechanism, amplification via the AP may account for more than 80% of all complement activation [19]. Certain SNPs in several complement components can predispose an individual to a more aggressive amplification loop, deficient regulation on target surfaces or a less prominent down regulation mechanism which is thought to affect the risk of developing AMD as well as disease severity. Supplementation of human serum with CFI was shown to dampen an over-activated complement system in high risk AMD sera by sequestering excess C3b via CFH binding (which outcompetes Factor B) and degradation of C3b required for AP C3 convertase formation [20]. It has therefore been considered that an increase in intra-ocular CFI level has the potential to dampen an over-activated complement system associated with AMD, reducing the progression of the disease. Using ocular delivery of CFI by gene therapy utilising Adeno-Associated Virus (AAV) vectors, sustained expression of human CFI in AMD patients and thereby reduction of local inflammation, might be achieved. This therapy is proposed to reduce ongoing inflammation at the site of the disease to slow down, pause or maybe even revert disease progression.

Here, the generation of AAV viral vector constructs carrying the cDNA of human CFI is described and successful in vitro and in vivo expression of active protein in human cell lines and murine retina is shown. These encouraging pre-clinical studies have led to a first-in-human AAV gene therapy trial for late-stage AMD (NCT03846193).

## Material and methods

### Codon optimisation and generation of plasmids

The cDNA of the most common human CFI sequence variant was downloaded from Genbank (Accession Number NM_000204.4) and ordered as gBlocks® Gene Fragments from Integrated DNA Technologies; this sequence is referred to as CFI. Four different codon optimised versions of the human CFI sequence were generated using publicly available codon optimising tools (GenScript, Gene Art, Integrated DNA Technologies and JCat); these codon-optimised (co) sequences are subsequently referred to as CFI.co-1, CFI.co-2, CFI.co-3 and CFI.co-4. All 5 constructs were further modified by insertion of a Kozak consensus sequence at the 5′-end. To generate pAAV.CFI, CFI cDNA sequences were cloned into a synthesised AAV genome backbone, consisting of 5′ and 3’ AAV2 inverted terminal repeats (ITR), a modified CAG promoter (comprised of a chicken β-actin promoter, a cytomegalovirus enhancer and a rabbit β-globulin intron, collectively termed CAG promoter) and a bovine growth hormone polyadenylation site. Additionally, a modified Woodchuck hepatitis virus post-transcriptional regulatory element (WPRE) was added downstream of the CFI cDNA sequence. WPRE was included to enhance short-term and long-term AAV-mediated transgene expression [21–23] and has been used in multiple clinical trials without any safety concerns [24, 25].

### Construction and production of AAV vectors

AAV vector was prepared by co-transfection of the HEK-293 (ATCC) cell line with pAAV.CFI or pAAV.CFI.co (pAAV.CFI.co-1 to pAAV.CFI-co.4) and pDG (Plasmid Factory), the latter providing adenoviral helper sequences and packaging sequences (Rep/Cap of AAV serotype 2). This serotype was chosen to enable strong transduction levels of the RPE and a lower level of transduction in the neurosensory retina. HEK-293 cells were grown in DMEM (Sigma) supplemented with 10% heat-inactivated foetal bovine serum (Gibco), 1% 200 mM L-Glutamine (Sigma Aldrich) and 1% Penicillin-Streptomycin (Sigma Aldrich). Cells were harvested after 72 h and lysed to purify the viral vector particles. These were purified using iodixanol gradient centrifugation. Vector was concentrated on Amicon Ultra-15 centrifugal filter units (Merck Millipore) and stored at −70 °C. Vector purity was assessed by SDS PAGE and the titre was determined by quantitative PCR.

### Transfections and viral transductions

All constructs were tested in vitro prior to in vivo expression studies in mice to confirm AAV, CFI transduction, expression and function. Cells were seeded in DMEM supplemented with 10% FBS at 1E + 06 (HEK-293) or 6E + 05 (ARPE-19) cells per well in a 6-well plate. Undifferentiated ARPE-19 cells were used for all experiments and an AAV carrying a GFP expression cassette was used as a negative control. For transfections, cells were incubated with 2.5 µg plasmid DNA and Lipofectamine LTX (Thermo Fisher Scientific) according to manufacturer’s recommendations. For transductions, cells were transduced at a MOI of 2.5E + 03 with each AAV construct for 16 h, after which cells were washed with PBS and incubated with fresh medium. Cells were incubated for a total of 72 h following plasmid or AAV delivery. Cells and supernatant were collected separately and stored at −70 °C. Cell pellets were divided for simultaneous analysis of protein and RNA expression.

### Subretinal injection

Female 8–10 week old C57BL/6 J mice (Charles River Laboratories) were used for all experiments. All animals were treated humanely in accordance with the UK Home Office Regulations under project license 30/3363. Mice were maintained on a 12:12-hour light/dark cycle. Mice were anaesthetised with a mixture of xylazine (10 mg/kg)/ketamine (80 mg/kg) in sterile saline; pupils were dilated with phenylephrine hydrochloride (2.5%) and tropicamide (1%). Proxymetacaine hydrochloride (0.5%) eye drops were used for additional local anaesthesia. An anterior chamber tap was performed prior to the subretinal injection using sterile 33 G needles (TSK Laboratory) and carbomer gel (Viscotears, Novartis Pharmaceuticals Ltd); and a small circular glass coverslip was used to achieve good visualisation of the fundus. The injection was performed through the posterior retina using a 10 μL NanoFil syringe and a 35 G bevelled NanoFil needle (World Precision Instruments Ltd). Anaesthesia was reversed with atipamezole (2 mg/kg) in sterile saline. Mice were injected with AAV.CFI or AAV.CFI.co-1 viral vector at two different doses (1E + 07 genome copies (gc)/eye [low dose] and 1E + 08 gc/eye [high dose]) in a 2 µL total volume. Prior to injection, vectors were diluted in 0.001% PF68 (Gibco) in PBS (Gibco). Sham injections were performed using diluent only. Per viral vector and dose group, 6 mice were injected in both eyes. After harvesting, eyes were dissected as follows: first, a posterior eye cup dissection was performed in 50 µL of PBS followed by retinal detachment from the RPE/choroid/sclera complex (referred to as “RPE” onwards). After retina and RPE tissue were isolated, both plus the remaining eyeball tissue were flushed with the dissection fluid enriched PBS. These isolated ‘wash’ sample and eye tissues were immediately transferred to dry ice and stored at −70 °C to prevent protein degradation. Tissues from three mice were pooled for protein expression analysis as described above.

### Reverse transcriptase quantitative PCR (RT-qPCR)

RNA was extracted from cell pellets (in vitro) or homogenised eye tissues (in vivo) using an RNeasy mini kit (Qiagen) as per manufacturer’s instructions. For quantification of CFI RNA, iTaq Universal SYBR Green Supermix (Bio-Rad) was used as per manufacturer’s instructions; primer sequences are shown in Table S1. Samples were run in a QuantStudio 6 Flex Real-time PCR system machine (Thermo Fisher Scientific). Relative expression of CFI (normalised to β-actin) was compared to the mean level in the corresponding control groups; the fold difference in expression was calculated using a qPCR efficiency corrected delta-delta-Ct approach [26].

### Immunoblotting

Cell pellets (in vitro) or homogenised eye tissues (in vivo) were resuspended in radioimmunoprecipitation assay (RIPA) buffer containing a proteinase inhibitor cocktail (Sigma). Tissues of 3 eyes were pooled to ensure detection of CFI protein. Protein concentrations of cell and tissue lysates were determined using a BCA protein assay kit (Thermo Scientific Pierce) as per the manufacturer’s instructions. Western blotting was performed by separation of proteins by means of SDS PAGE, followed by semi-dry blotting and immunostaining of CFI, GFP or β-actin bands. Equal volumes of cell culture supernatants were loaded and for lysates, normalised amounts of protein, determined by BCA protein assay, were loaded for each group. CFI was detected with a mouse monoclonal antibody to human CFI (1:500 dilution, OX21, Thermo Fisher Scientific) or a goat anti-human CFI (1:1000 dilution, Comptech, A238). GFP was detected with rabbit polyclonal antibody to TurboGFP (1:1000 dilution, Thermo Fisher Scientific, PA5-22688) and β-actin was detected with mouse anti-β-actin (1:10000 dilution, Thermo Fisher Scientific, AM4302). Protein bands were visualised using Clarity Western ECL Substrate (Bio-Rad) and analysed with an Odyssey® Fc Imaging System. Immunoblots of transfected cells were analysed using Image J software. In brief, bands were selected as region of interest, plotted as histograms and the area under the curve was calculated for each lane.

### Preparation of retinal sections for immunohistochemistry

Eyes were removed and a hole was made just behind the ora serrata. The eyeball was placed in 4% (w/v) paraformaldehyde in 0.12 M phosphate buffer, pH 7.2 for 1 hour at room temperature. Next, the anterior segment and the lens were removed, and the eyes were prepared for sections or whole mount. Eye cups used for sections were cryopreserved with increasing concentrations of sucrose rxin 0.12 M phosphate buffer, pH 7.2 (10%, for 1 h at room temperature and 30% overnight at +4 °C), embedded in OCT (Tissue-tek) and frozen in a mould at −70 °C. Sections were cut at a thickness of 10 µm on a cryostat and mounted onto glass slides (Super-Frost, Thermo Fisher Scientific). The slides were air dried for 2 h at room temperature and stored at −70 °C. For eye cups used for whole mounts, retina and RPE/choroid were separated by gently peeling the retina off the RPE and stored separately in PBS at +4 °C.

### Immunostaining

Sections were blocked and permeabilised by incubation at room temperature for 60 min in PBSGT solution (0.2% (w/v) gelatine, 0.25% (v/v) Triton X-100 in PBS). Whole mounts were blocked and permeabilized by incubation at room temperature for 2 h in 0.2% (w/v) gelatine, 1.5% (v/v) Triton X-100 in PBS. Subsequently, sections and whole mounts were incubated with primary antibodies diluted in PBSGT solution overnight at room temperature. CFI was detected with rabbit anti-CFI (1:250 dilution, Sigma Aldrich, HPA024061) and phalloidin (to visualise actin filaments and cytoskeleton) was stained with Alexa Fluor 488 phalloidin (1:5,000, Life Technologies, A12379). After washing in PBST solution (0.1% (v/v) Triton X-100 in PBS), sections and whole mounts were incubated with goat anti-rabbit secondary antibody coupled to Alexa Fluor594 (1:5000, Life Technologies, Thermo Fisher Scientific, A32731) and stained with DAPI in PBSGT solution for 1.5 h at room temperature. The slides were washed with PBST solution and subsequently cover-slipped with mounting medium (Mowiol, Merck Millipore).

### C3b cleavage assay

A C3b cleavage assay was performed to confirm functional activity of CFI expressed after subretinal injection in mice. In this assay, the C3 alpha chain is degraded by CFI into iC3b which can be detected by immunoblotting as a reduction of the alpha chain at 116 kDa and appearance of the two iC3b breakdown bands at 68 and 43 kDa. 20 µL of dissection ‘Wash’ sample collected during dissection of eyes was incubated with 1 µg purified C3b (Complement Technology Inc.) and 0.5 µg purified CFH (Complement Technology Inc.) for 1 hour at 37 °C. The same volume of ‘wash’ sample was standardised amongst all in vivo study conditions to enable accurate comparison of FI functional activity. C3b cleavage products were detected by western blotting using a goat anti-human C3 antibody (1:5000 dilution, AbD Serotec/BioRad, AHP1752) and Peroxidase conjugate AffiniPure Donkey Anti-goat IgG (1:5000, Jackson ImmunoResearch, 705-035-147).

### Statistical analysis

Statistical analysis was performed using GraphPad Prism 8.4.0. If data were normally distributed with equal variance then parametric tests were used (ANOVA) with Tukey’s or Sidak’s multiple comparisons tests (Dunnett’s for data sets with unequal variance). If data were not normally distributed, then non-parametric tests were performed (Kruskal-Wallis) with Dunn’s multiple comparison test. Analyses performed for each data set are stated in the respective figure legends.

## Results

### Generation of 5 AAV.CFI viral vector constructs

Five AAV viral vectors were generated carrying either the cDNA sequence of the human CFI gene or codon optimised versions of the CFI sequence. A schematic diagram of the vectors is shown in Fig. 1B. Codon optimised sequences of CFI cDNA were generated using 4 different online tools as described in methods. The 1752 bp CFI sequence was placed under the control of the ubiquitous CAG promoter, followed by a WPRE and a bovine growth hormone poly-A site with AAV2 ITRs at each end (Fig. 1B).

### CFI is expressed from human cell lines after transfection and transduction

**Fig. 2:**
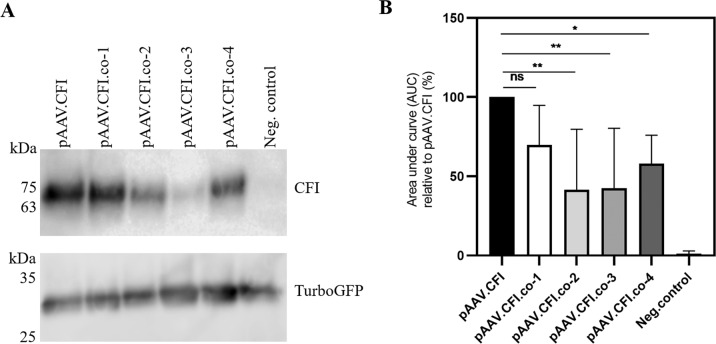
Immunoblot analysis of supernatants and cell lysates of pAAV.CFI, pAAV.CFI.co and pCMV.GFP co-transfected ARPE-19 cells. **A** Upper immunoblot: Supernatants of transiently transfected cells. CFI and codon-optimised CFI were detected with mouse anti-human CFI. Plasmid backbone containing AAV sequences without transgene served as a negative control (representative result). Supernatant was loaded non-reduced to prevent separation of heavy and light chain. Lower immunoblot: Lysate of transiently transfected cells. GFP was detected with anti-turbo-GFP and implies that there were not large differences in transfection efficiency between the groups. **B** Quantification of CFI expression immunoblots by densitometry analysis using ImageJ software. Equal volumes were loaded in each experiment and summary data from 7 independent experiments is shown. Results are presented as relative density of CFI signal relative to signal intensity of AAV.CFI.wt. Data are shown as mean + standard deviation. AAV = adeno-associated virus, CFI = complement factor I, co = codon optimised. Asterisks represent significant differences between codon optimised constructs and the most common CFI sequence (reference) analysed by one-way ANOVA and Dunn’s multiple comparison test: * = *P* < 0.05, ** = *P* < 0.01, ns = not significant.

Generated pAAV.CFI and pAAV.CFI.co-1 to pAAV.CFI.co-4 plasmid constructs were transfected into the ARPE-19 cell line to confirm expression of CFI. Cells were co-transfected with a Turbo-GFP plasmid to ensure that cells were equally transfected. CFI was expressed from each construct and protein was detected as a band at 63 kDa (Fig. 2A, upper). Turbo-GFP staining, as a transfection control, was similar in all transfected cells (Fig. 2A, lower). Total CFI expression varied across constructs tested and only one codon-optimised construct, pAAV.CFI.co-1, yielded CFI concentrations similar to the wild-type CFI sequence, pAAV.CFI. The other three constructs resulted in significantly less CFI protein (Fig. 2B).

pAAV.CFI and pAAV.CFI.co-1 were selected and packaged into AAV2 capsids. HEK-293 and ARPE-19 cells were transduced with AAV.CFI and AAV.CFI.co-1 at an MOI of 1E + 04. CFI protein expression and secretion were confirmed by immunoblotting and again did not show differences in secreted levels of CFI (Figure S2).

### CFI is secreted from RPE cells and functionally active after subretinal injection of AAV.CFI constructs

**Fig. 3:**
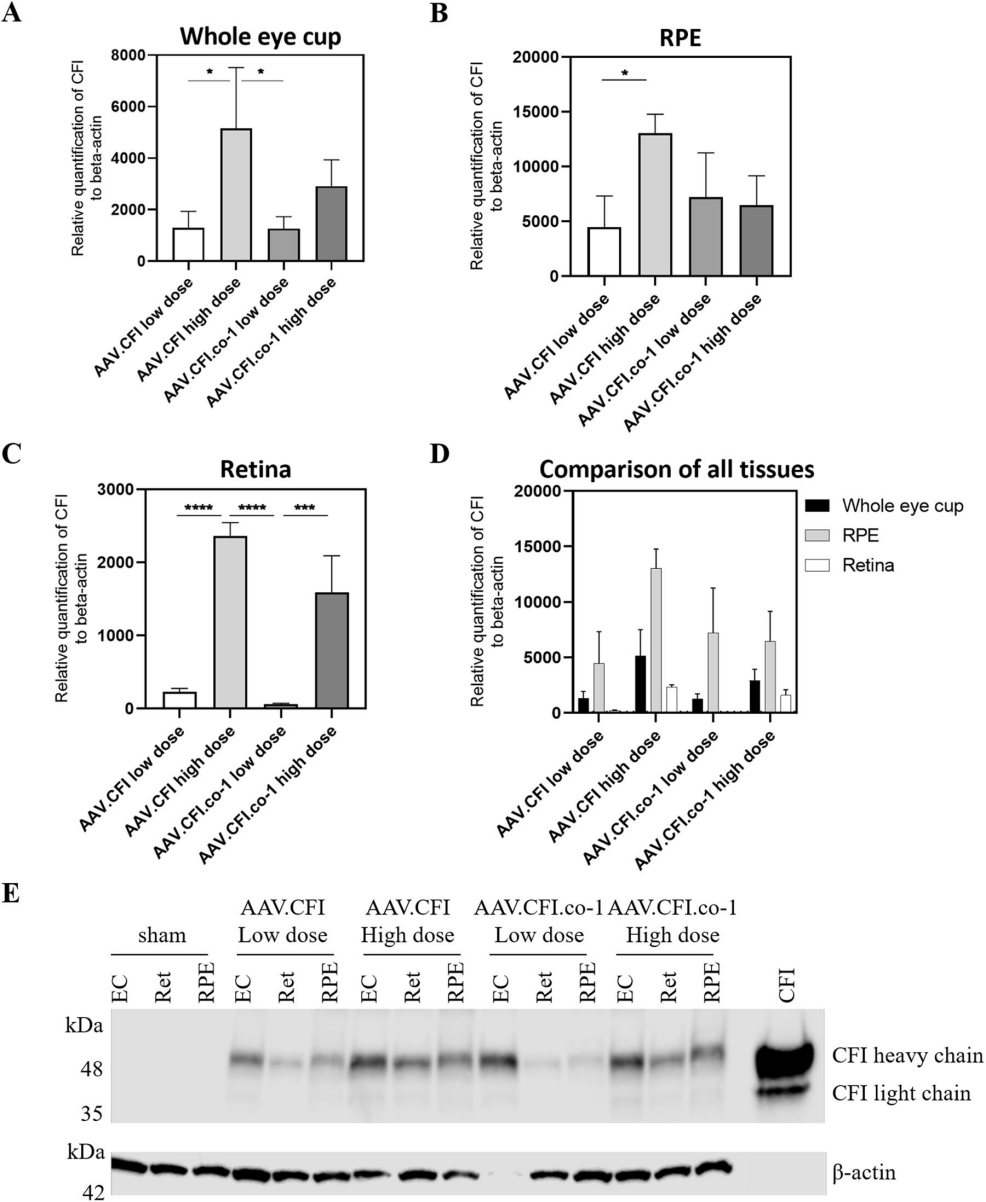
In vivo expression of CFI RNA and protein. **A**–**D** RT-qPCR to analyse gene expression of AAV.CFI and AAV.CFI.co-1 (*n* = 3 per condition) of whole eye cup (**A**), RPE (**B**), retina (**C**), and a comparison of all tissues analysed (**D**). RPE samples generated more CFI mRNA than retina (*p* < 0.0001) and multiple comparisons revealed significant differences between injection materials, which are described in the text. Data are shown as mean + standard deviation. **E** Immunoblot of whole eye cups, retina, and RPE of mice injected with AAV.CFI.wt or AAV.CFI.co-1, samples of three mice per condition were pooled for analysis. β-actin staining was performed as a loading control. AAV = adeno-associated virus, CFI = complement factor I, co = codon optimised, ec = eye cup, ret = retina, RPE = retinal pigment epithelium. Asterisks represent significant differences between constructs analysed by one-way ANOVA with Tukey’s multiple comparison: * = *P* < 0.05, ** = *P* < 0.01, *** = *P* < 0.001, **** = *P* < 0.0001.

Female 8 to 10-week-old C57BL/6 J mice received a subretinal injection via the transscleral route with two doses (1E + 07 [low dose] or 1E + 08 vg/eye [high dose]) of AAV.CFI or AAV.CFI.co-1 and CFI expression was analysed after 4 weeks. The expression of CFI in lysates of mouse retinal tissues was confirmed by RT-qPCR analysis. The tissue type, dose and injection material had a significant influence on the relative mRNA levels of CFI. A strong dose effect was observed in AAV.CFI in all tissues analysed (Fig. 3A-D); however, a significant difference between high and low dose of AAV.CFI.co-1 was only detected in the retina (Fig. 3C). The RPE provided significantly more relative CFI mRNA than other retinal tissue (Fig. 3D). No CFI expression was detected in the control injected samples (data not shown).

Western blotting of lysed tissue fractions revealed CFI protein with bands detected at 50 kDa (heavy chain) and 30 kDa (light chain) (Fig. 3E). Both AAV constructs resulted in expression of CFI protein at each dose. Expression of CFI was detected in all sample types and a trend for higher expression in the eye cup sample was observed when band intensity was compared visually. No human CFI was detected in the control injected eyes.

**Fig. 4:**
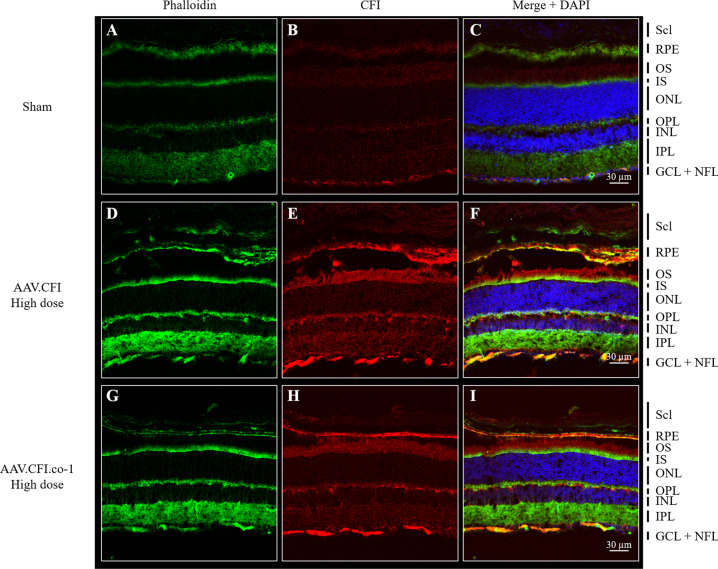
Immunohistological analysis of retinal sections of AAV.CFI and AAV.CFI.co-1 injected mouse eyes. 10 µm retinal sections of sham (**A**–**C**), AAV.CFI (**D**–**F**) or AAV.CFI.co-1 (**G**–**I**) injected mice stained with anti-phalloidin, DAPI, and anti-CFI to demonstrate that CFI protein localises to the RPE cell layer and photoreceptors. AAV = adeno-associated virus, CFI = complement factor I, co = codon optimised, GCL = ganglion cell layer, IS = inner segment of photoreceptors, NFL = nerve fibre layer; ONL = outer nuclear layer, OPL = outer plexiform layer, OS = outer segment of photoreceptors, RPE = retinal pigment epithelium, Scl = Sclera. Magnification: ×20.

Immunohistological analysis of eye cups was performed to confirm RPE expression and to determine in vivo location of CFI. Phalloidin and DAPI co-staining were used to distinguish the different retinal layers. CFI protein was detected in eyes of mice injected with AAV.CFI and AAV.CFI.co-1 and localised mainly in two compartments: strong expression was detected in the RPE and weaker expression in both the outer and inner segments of photoreceptors (Fig. 4 to Fig. 6 and Figure S3 to Figure S5). Wholemount en face view of the posterior eye cup showed expression of CFI protein in the hexagonal RPE cells (Fig. 6). The staining appeared granular, which could indicate localisation of CFI within secretory pathway vesicles. Staining intensity and location detected in AAV.CFI and AAV.CFI.co-1 injected mouse eye retinal sections was similar, although CFI immunoreactivity appeared to be more punctate in AAV.CFI (Fig. 4F) and more linear (Fig. 4I) in AAV.CFI.co-1 injected eyes staining at the junction of OPL and INL retinal layers.

**Fig. 5:**
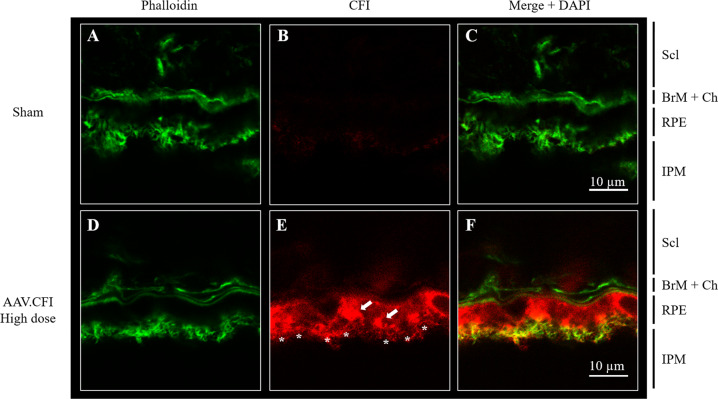
Immunohistological analysis retinal sections of sham and AAV.CFI injected mouse eyes – higher magnification of RPE. Immunohistological analysis retinal sections of sham (**A-C**) and AAV.CFI (**D-F**) injected mouse eyes. Retinal sections were double labelled with phalloidin to stain actin filaments (**A**, **D**) and CFI (**B**, **E**). Nuclei were stained with DAPI and are shown in merge (**C**, **F**). Vesicular staining is depicted with arrows, RPE microvilli are highlighted with asterisk. AAV = adeno-associated virus, BrM: Bruch’s membrane, CFI = complement factor I, Chr: choriocapillaris, IPM: inter photoreceptor matrix, RPE: retinal pigment epithelium, Scl: sclera. Magnification: 189x.

**Fig. 6:**
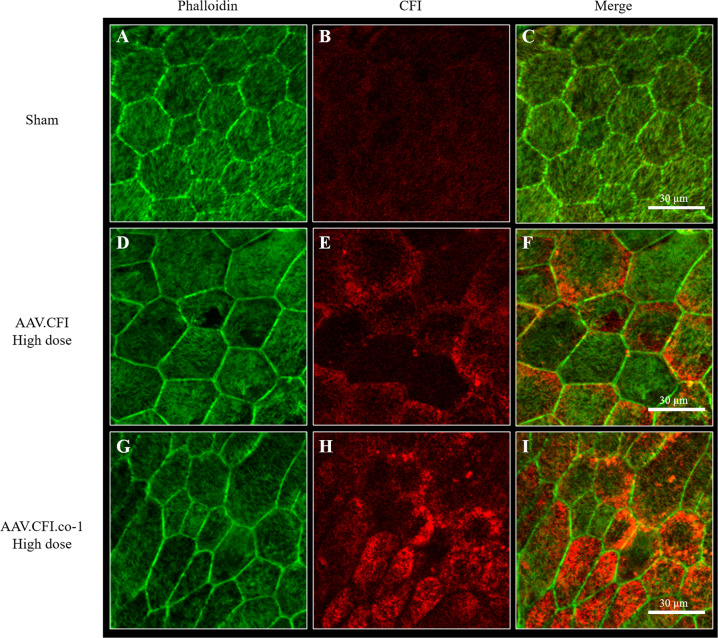
Immunohistological analysis of whole mounted RPE of AAV.CFI and AAV.CFI.co-1 injected mouse eyes. Whole mounted RPE of sham (**A-C**), AAV.CFI (**D-F**) and AAV.CFI.co-1 (**G-I**) injected mice was stained with anti-phalloidin and anti-CFI. CFI is highly expressed in RPE cells of AAV.CFI.wt and AAV.CFI.gs treated mice. Abbreviations: AAV = adeno-associated virus, CFI = complement factor I, co = codon optimised. Magnification: 40x.

**Fig. 7:**
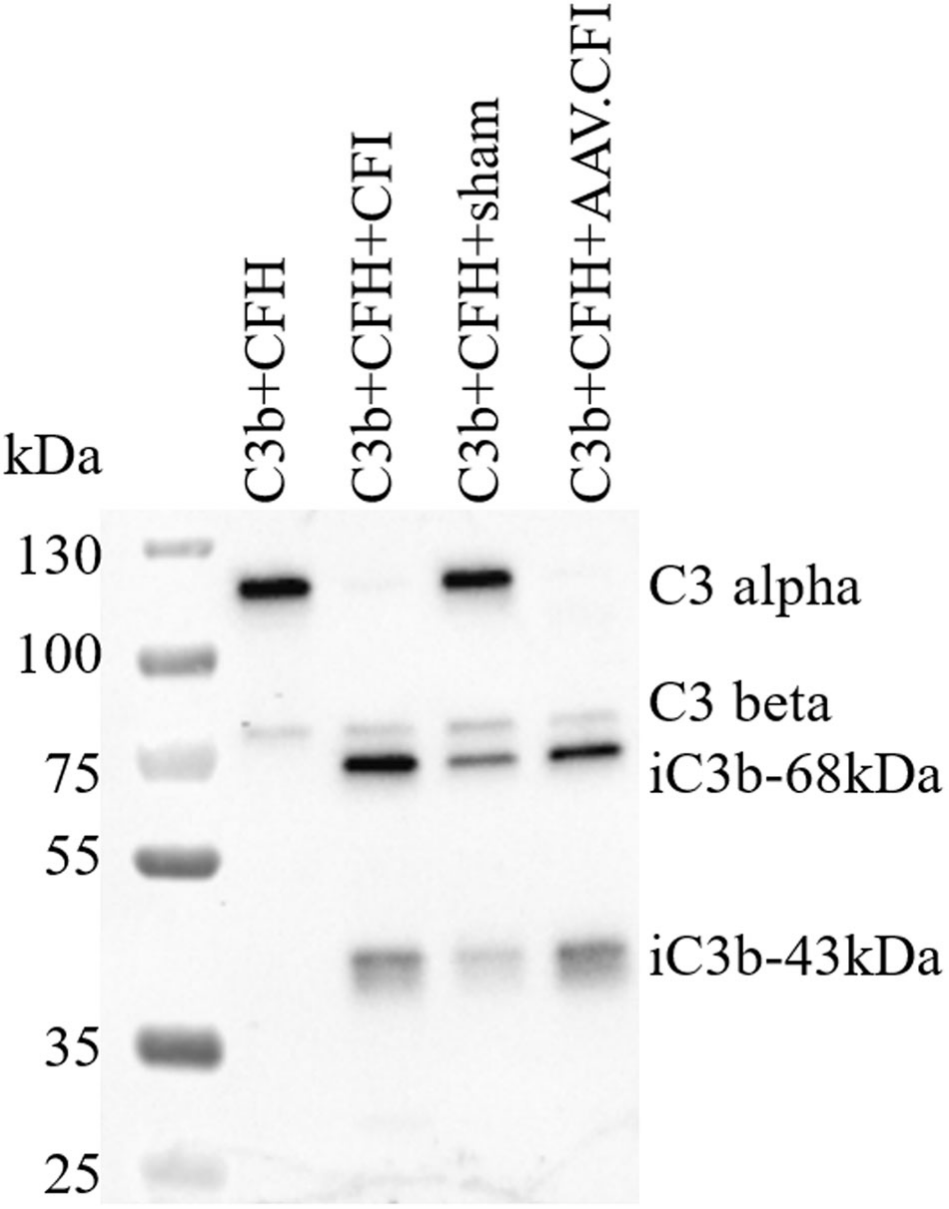
Ex vivo demonstration of CFI functional activity by a cofactor assay following subretinal administration. C3b, the substrate, is incubated with CFH as a cofactor; in the absence of CFI no degradation occurs (lane ‘C3b + CFH’, assay negative control). As soon as C3b is incubated with CFH and CFI, degradation of C3b into iC3b occurs, visible as 68 and 43 kDa bands (lane ‘C3b + CFH *+* CFI, assay positive control). C3 and CFH are incubated with ‘wash’ sample derived from either sham injected or AAV.CFI injected mice as a source of CFI and degradation of the C3 alpha band into the iC3b bands is observed. Abbreviations: AAV *=* adeno-associated virus, C3 *=* complement C3, CFI *=* complement factor I, CFH *=* complement factor H.

Due to the small volume in mice, it is not easy to directly analyse the vitreous humour for presence and processing of secreted CFI protein. During dissection of eyes, tissues were rinsed with PBS and used as “wash” sample for functional testing of CFI protein by a C3b cleavage assay. Presence and activity of CFI is measured by degradation of C3b and detected by immunoblotting. Figure 7 shows that control injected “wash” samples have low C3b processing activity, as evidenced by only partial degradation of the C3 alpha chain into 68 and 43 kDa iC3b bands, likely due to low levels of endogenous mouse CFI expression. AAV.CFI injected “wash” samples showed increased C3b degradation and complete degradation of the C3 alpha band into the iC3b breakdown bands, demonstrating that CFI was both secreted and functional (Fig. 7, only AAV.CFI was analysed in this assay).

## Discussion

This paper describes the development and characterisation of five vectors for a gene therapy to treat AMD. These results show for the first time, in vivo expression of active human CFI protein in mice. By subretinal injection, it is possible to target the majority of AAV.CFI constructs to the RPE which sits between Bruch’s membrane and photoreceptors. CFI is a complement regulator that only through post-translational modification is converted from a single polypeptide chain (pro-CFI) into the dimeric active enzyme. Furin cleaves pro-CFI into a heavy and a light chain [27] and only then is CFI able to bind to the C3b-FH complex and orientate its light chain containing the serine protease domain to enable cleavage of C3b into iC3b [28]. In vitro expression of CFI often results in incomplete processing probably due to insufficient furin processing activity [27] and therefore reduced activity of recombinant CFI protein. Here, over-expression of CFI protein in mouse retinas resulted in fully active CFI that is able to cleave its natural substrate C3b into iC3b when tested ex vivo.

Of note, western blot and RT-qPCR analysis do not correlate with respect to which tissue has the highest CFI expression; however, a dose-effect is observed in both analyses with AAV.CFI resulting in higher CFI expression levels than AAV.CFI.co-1. The reason for the discrepancy between both methods could be low sample numbers and the fact that three tissue samples were pooled for immunoblot analysis.

Codon optimisation as employed in this study is often used to increase levels of recombinant proteins. Most amino acids are encoded by multiple synonymous codons but the relative expression of individual tRNAs can vary dramatically across species and even cell or tissue types [29]. Codon optimisation tools aim at maximising expression rates by either adjusting codon usage to resemble the natural distribution of the host organism by removing rare codons in exchange for more frequently used codons or by avoiding occurrences of codon-pairs that are known to translate slowly (reviewed in [30]). Rapid translation is, however, not always beneficial and it is thought that translation sometimes is slowed down by rare codon usage to enable correct folding of certain protein domains [31]. Therefore, codon optimisation does not always lead to higher protein expression, as shown in this study, and highlights our limited understanding of codon usage in transgene expression. It is also noteworthy that, although correct protein sequence will be retained, SNPs introduced due to alternative codon usage can result in unanticipated alteration of RNA sequences by removal of pre-existing and/or introduction of novel editing sites [30], thereby creating novel or even pathogenic gene sequences. All downstream effects of a codon optimised sequence should therefore be critically evaluated before permanent and sustained expression by gene therapy to avoid potentially serious consequences. In this study, none of the four tested codon optimised sequences were found to enhance expression in vitro and in vivo.

Current drug development efforts and clinical studies for the treatment of AMD and/or geographic atrophy focus on inhibition of the complement system, anti-inflammatory drugs, neuroprotection, reduction of toxic by-products, improvement of mitochondrial function, and stem cell therapies [31]. After a first wave of drug candidates targeting the complement system failed to meet clinical endpoints in AMD and/or geographic atrophy (for example Lampalizumab [Genentech Inc., Roche, NCT02247479 and NCT02247531], Eculizumab [Alexion Pharmaceuticals Inc., NCT00935883], LFG316 [Novartis Pharmaceuticals], LFG316 [Novartis Pharmaceuticals, NCT02515942]), a plethora of new therapeutic approaches are currently in clinical development [32]. The exact reasons for the failure of previously tested complement therapeutics are unknown. However, it is likely to be a combination of the chosen target proteins, mode of delivery, nature of drug, insufficient levels for protection or unfavourable diffusion properties within the eye and through Bruch’s membrane. Alternatively, it is also possible that intervention by targeting the complement system is less efficient in geographic atrophy and would need to be applied during earlier stages of the disease. Overall, they also underline the fact that there is currently still a limited understanding of how complement activation is regulated in AMD. Over the last year; however, positive results of phase 2 interventional trials have been announced which support the complement system as a drug target in the treatment of geographic atrophy: APL, a C3 inhibitor developed by Apellis Pharmaceuticals, Inc (NCT03525600 and NCT03525613) and Zimura, a C5 inhibitor, developed by Iveric bio, formerly Ophthotech Corporation (NCT02686658). Overexpression of CFI in the eye has the potential of restoring local complement activation accompanying AMD and thereby slowing down progression of atrophy. In contrast to other complement inhibitors which aim to completely abolish complement activation, increasing levels of CFI will re-balance the complement system to prevent excessive activation whilst maintaining low levels of complement turnover required to maintain homoeostasis in the eye [33]. As shown by Lachmann et al. [20], an increase in plasma concentration of CFI by 50% is sufficient to protect serum from donors with an AMD at-risk genetic composition from complement hyperactivation. In the absence of an approved therapeutic treatment of late stage AMD, local CFI supplementation might have the potential to slow down disease progress by limiting complement mediated inflammation in the eye. The data presented here led to the formulation of the GT005 product which is now under evaluation in a first-in-human clinical trial delivering an AAV gene therapy for late stage AMD (Gyroscope Therapeutics Ltd, NCT03846193).

## Supplementary information

Supplementary Material
