# Supplementary material for: Functional expression of complement factor I following AAV-mediated gene delivery in the retina of mice and human cells
Source: Gene Ther. 2021 Mar 10;28(5):265–76. doi: 10.1038/s41434-021-00239-9 (PMC8149295; doi:10.1038/s41434-021-00239-9)
Supplement: Supplementary file 1 — Supplementary Material [file 41434_2021_239_MOESM1_ESM.docx]

**Supplementary Material**

**Figure S 1**


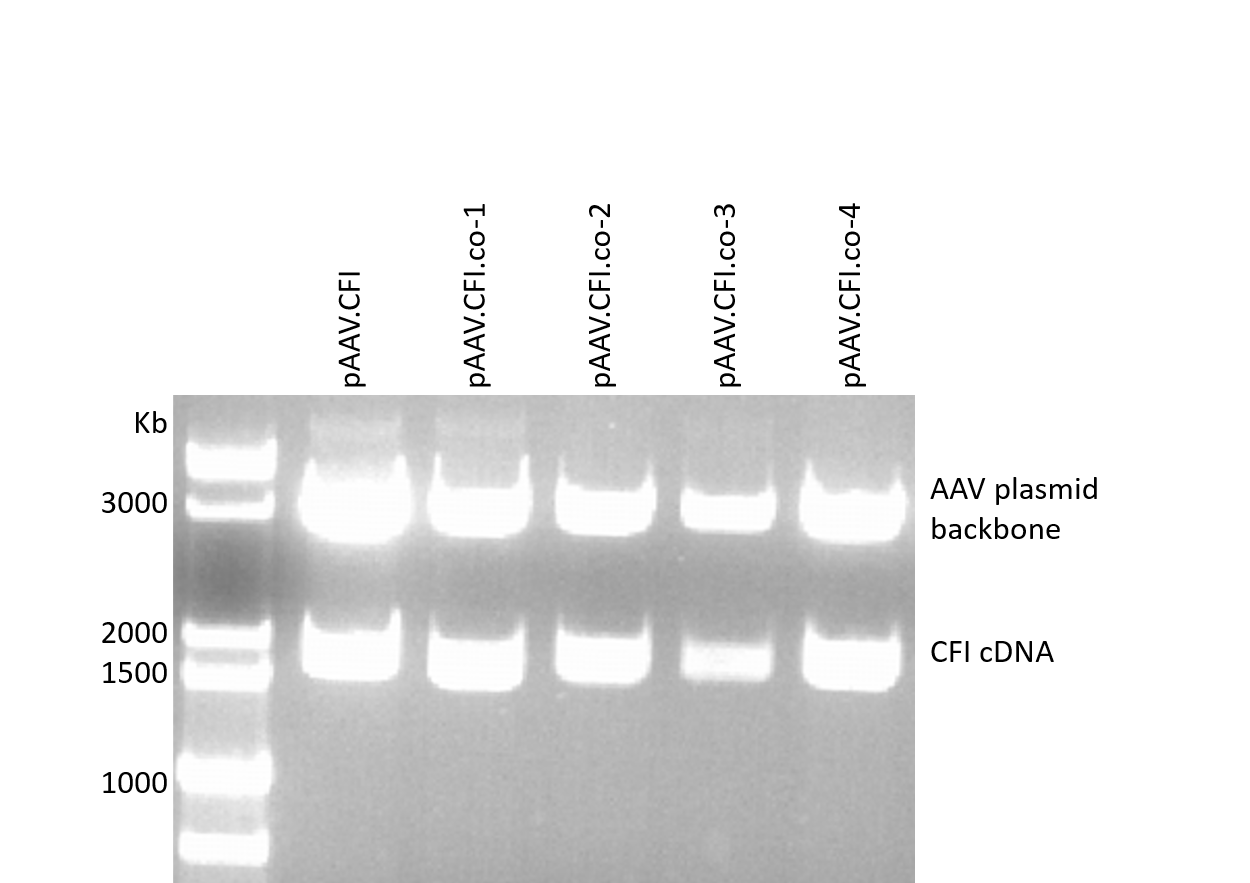


Agarose gel showing restriction digests of generated AAV.CFI viral vectors. CFI cDNA is 1,752 bp. Abbreviations: AAV = adeno-associated virus, CFI = complement factor I, co = codon optimised

**Table S 1**

| **Primer** | **Sequence** |
| --- | --- |
| CFI_F | GGATTTCGCTGATGTGGTTT |
| CFI_R | CCCTGTAATGCAGTCCACCT |
| CFI.co-1_F | GCAGAGTGCACCTTCACAAA |
| CFI.co-1_R | TGACAGGCCTTACAGCACAG |
| Actin_F | CTTCTGGCATCCTGTCAGCAA |
| Actin_R | CCTGGGTATGGAATCCTGTG |

Primer sequences used for quantification of CFI expression. Abbreviations: CFI = complement factor I, co = codon optimised, F = forward primer, R = reverse primer

**Figure S 2**


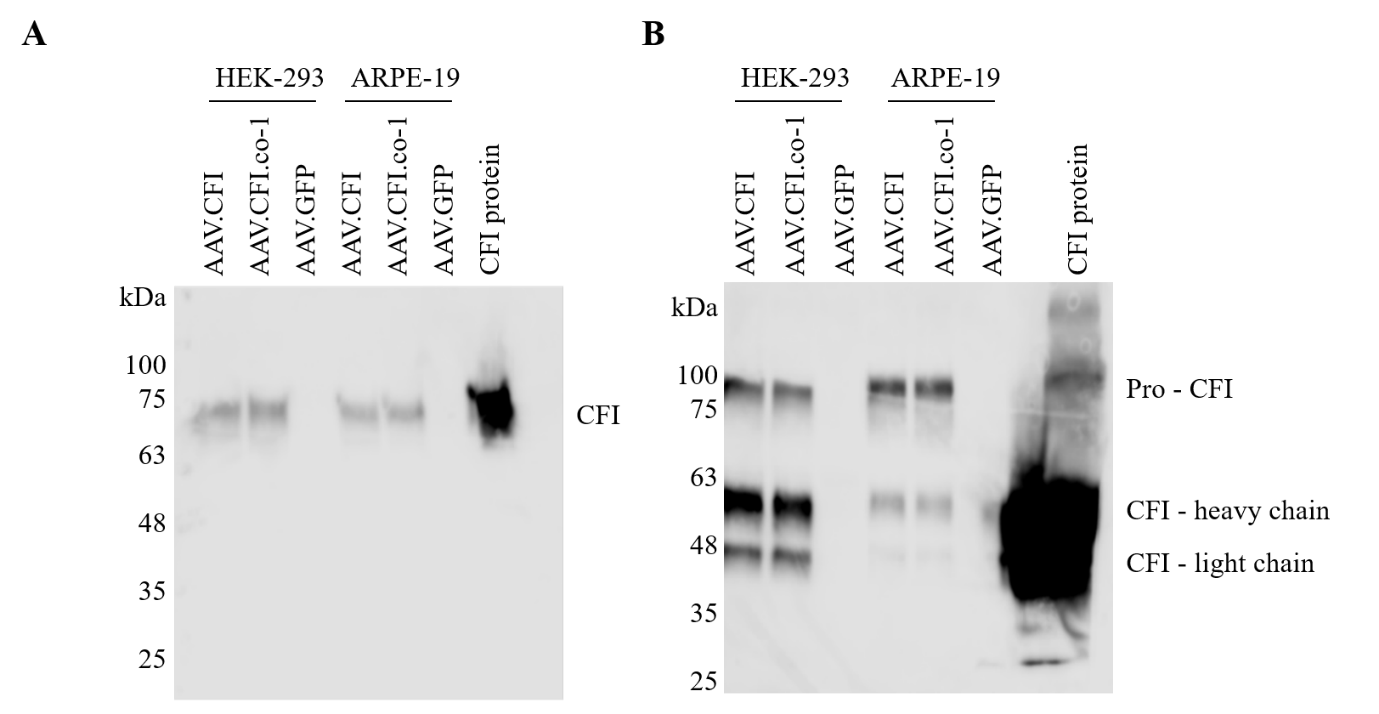


Transduction of HEK-293 and ARPE-19 cells with AAV constructs. **A**: Non-reduced immunoblot shows CFI protein as a single chain. **B**: Under reducing conditions CFI is detected as pro-CFI, heavy and light chain. AAV.GFP is used as a negative control, purified CFI protein is used as a positive control. Abbreviations: AAV = adeno-associated virus, CFI = complement factor I, co = codon optimised

**Figure S 3**


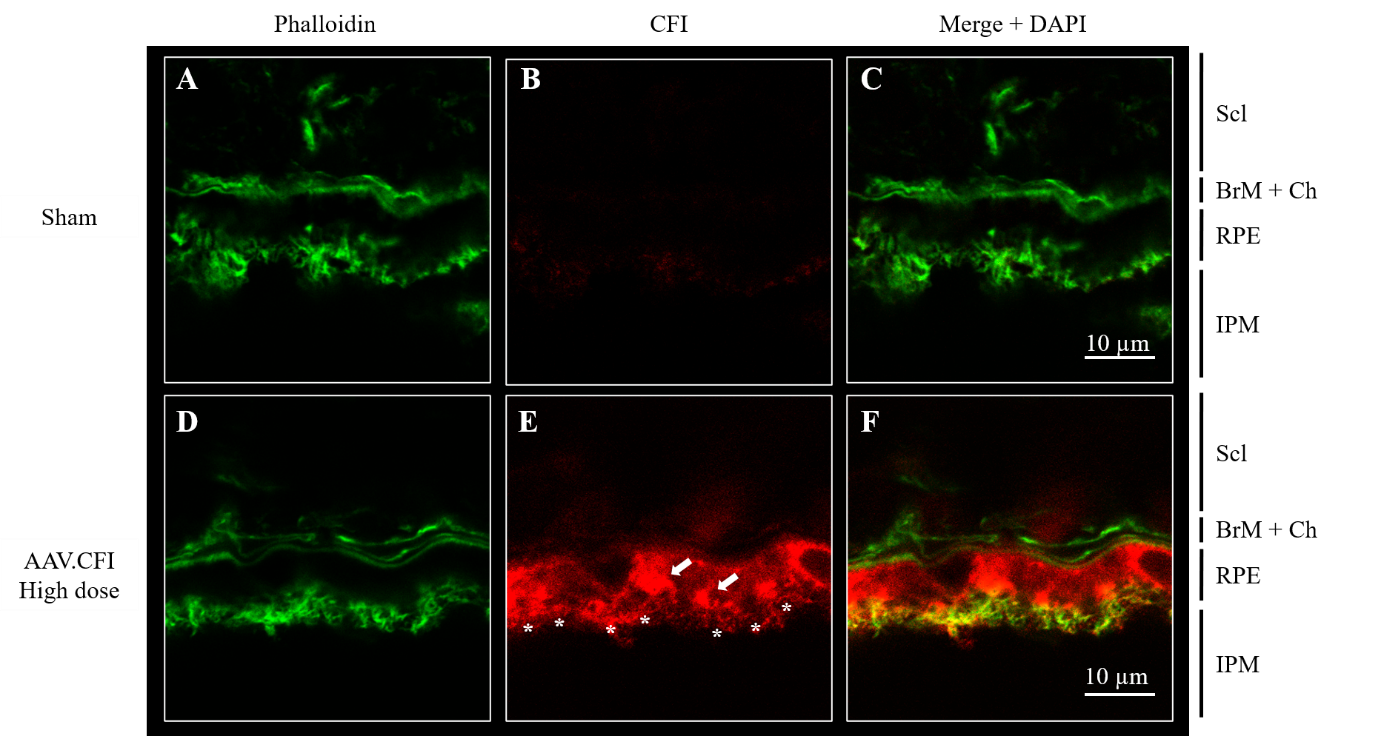


Immunohistological analysis retinal sections of sham (**A-C**) and AAV.CFI (**D-F**) injected mouse eyes – higher magnification of the photoreceptor layer. Retinal sections were double labelled with phalloidin to stain actin filaments in the inner segment of photoreceptors (**A** and **D**) and CFI (**B** and **E**). Nuclei were stained with DAPI and are shown in merge (**C** and **F**). CFI expression in AAV.CFI.wt injected eyes is detected in the outer segment of photoreceptors. Abbreviations: AAV = adeno-associated virus, CFI = complement factor I, IPM= inter photoreceptor matrix, IS = inner segment, ONL = outer nuclear layer, OS = outer segment. Magnification: 189x

**Figure S 4**
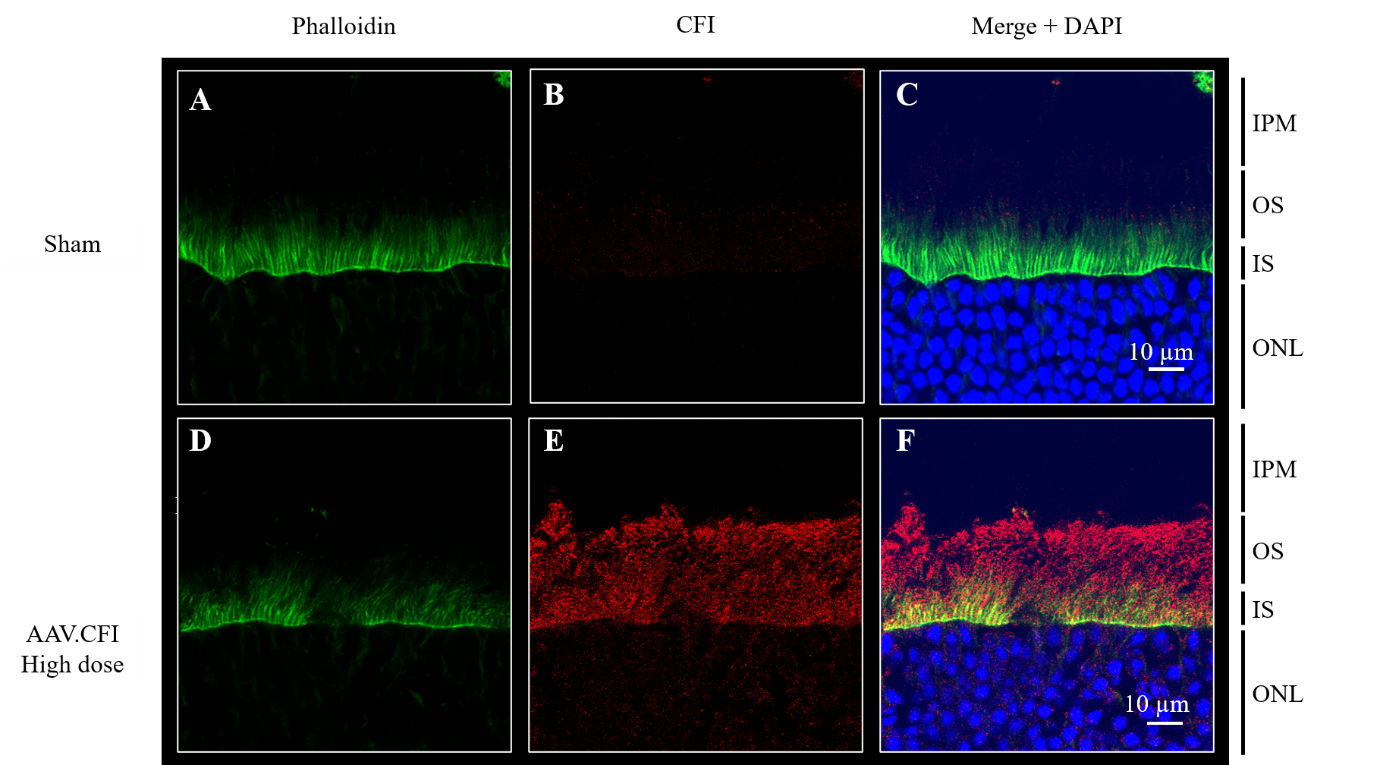


Immunohistological analysis retinal sections of sham (**A-C**) and AAV.CFI (**D-F**) injected mouse eyes – higher magnification of the outer plexiform layer. Retinal sections were double labelled with phalloidin to stain actin filaments (**A** and **D**) and hCFI (**B** and **E**). Nuclei were stained with DAPI and are shown in merge (**C** and **F**). Horizontal cells staining is depicted with arrows. Abbreviations: AAV = adeno-associated virus, CFI = complement factor I, INL = inner nuclear layer, ONL = outer nuclear layer, OPL = outer plexiform layer. Magnification: 189x

**Figure S 5**


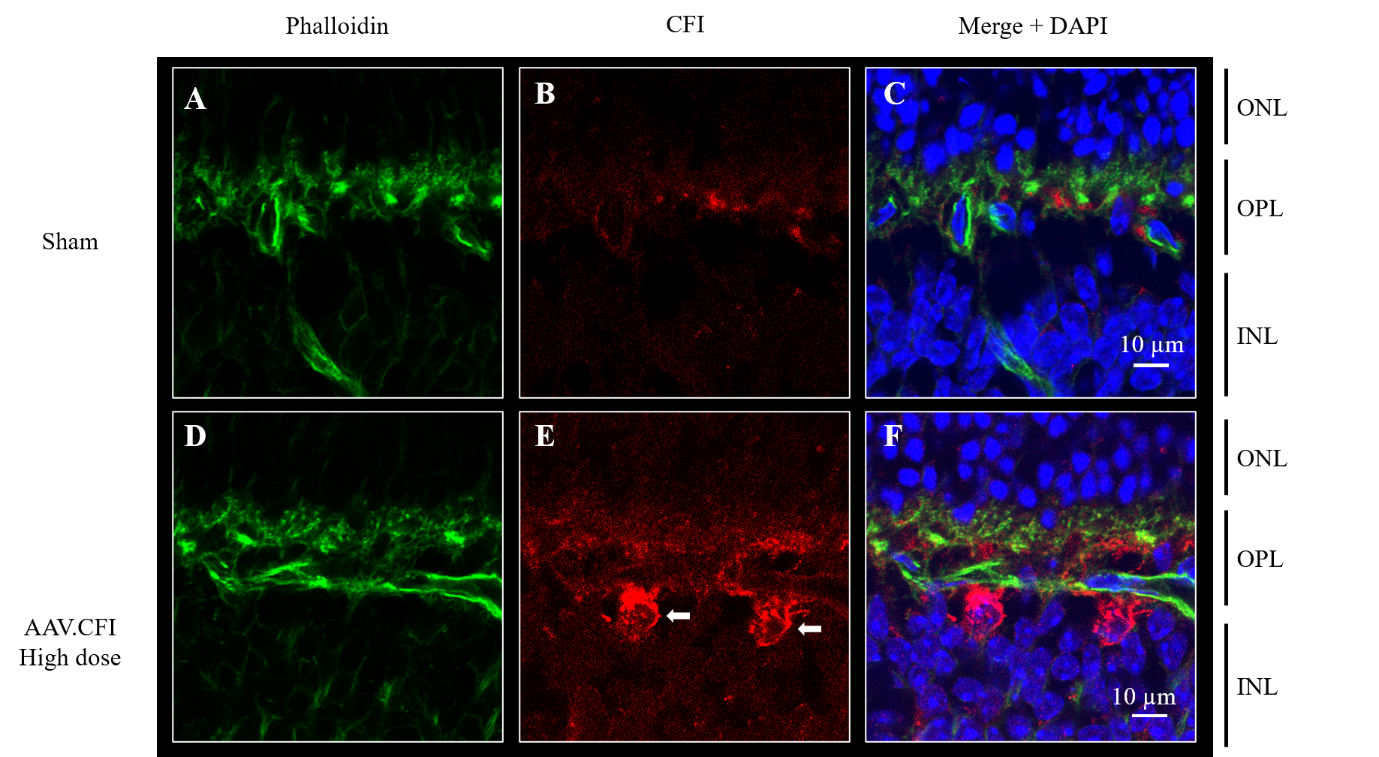


Immunohistological analysis retinal sections of sham (**A-C**) and AAV.CFI (**D-F**) injected mouse eyes – higher magnification of the ganglion cell layer. Retinal sections were double labelled with phalloidin to stain actin filaments (**A** and **D**) and CFI (**B** and **E**). Nuclei were stained with DAPI and are shown in merge (**C** and **F**). Abbreviations AAV = adeno-associated virus, CFI = complement factor I, GCL = ganglion cell layer, IPL: inner plexiform layer, NFL = nerve fibre layer. Magnification: 189x
